# Health Habits of Employees in a Large Medical Center: Time Trends and Impact of a Worksite Wellness Facility

**DOI:** 10.1038/srep20804

**Published:** 2016-02-11

**Authors:** Abd Moain Abu Dabrh, Archana Gorty, Sarah M. Jenkins, Mohammad Hassan Murad, Donald D. Hensrud

**Affiliations:** 1 Division of Preventive, Occupational, and Aerospace Medicine, Mayo Clinic, Rochester, MN, USA; 2 Department of Biomedical Statistics and Informatics, Mayo Clinic, Rochester, MN, USA

## Abstract

Worksite health interventions are not novel but their effect remains subject of debate. We examined employer-based wellness program to determine health habits trends, and compare prevalence estimates to national data. We conducted serial surveys (1996 and 2007–10) to employees of a large medical center that included questions measuring outcomes, including obesity, regular exercise, cardiovascular activity, and smoking status. Logistic regression models were estimated to compare data by membership across years, considering p-values ≤ 0.01 as statistically significant. 3,206 employees responded (Response rates 59–68%). Obesity prevalence increased over time in members and nonmembers of the wellness facility, consistent with national trends. Members had a lower prevalence of cigarette smoking compared to nonmembers (overall year-adjusted odds ratio 0.66, P < 0.001). Further, employees had a lower prevalence of cigarette smoking (9.7 vs. 17.3% in 2010, P < 0.001) compared with national data. Wellness facility membership was associated with increased regular exercise and cardiovascular exercise (P < 0.001) compared to nonmembers. In summary, working in a medical center was associated with a decreased prevalence of cigarette smoking, but not with lower prevalence of obesity. Worksite wellness facility membership was associated with increased exercise and decreased cigarette smoking. Employer-based interventions may be effective in improving some health behaviors.

Escalating healthcare costs and health problems such as obesity, physical inactivity, and poor dietary habits are among the top health issues facing the nation today, possibly leading to a reduced average lifespan for the first time since the 1900s^1^. National surveillance data indicate that many Americans continue to engage in suboptimal health habits that increase the risk of associated chronic diseases. Most full-time employees spend at least 50 hours per week at work and consume about a third of their meals at work^2^. Therefore, the workplace is well-suited as a venue to intervene and improve personal health habits. In fact, as part of the Healthy People 2020 initiative, the United States Department of Health and Human Services (USDHHS) announced a goal whereby 75% of all worksites in the United States will offer comprehensive health promotion program opportunities to employees. A corollary goal involves the engagement of 75% of employees at each worksite as participants in health promotion programs^3^.

Worksite health interventions such as wellness programs, fitness facilities, and educational programs are not novel. Such interventions have shown effectiveness in decreasing various health risk factors, increasing productivity, minimizing short-term absenteeism, and even decreasing employee’s health care costs^4^,^5^,^6^,^7^. In assessing employees in a health care setting, the Behavioral Risk Factor Surveillance System (BRFSS), which included more than 21,000 healthcare workers, found that healthcare workers have a prevalence of smoking and obesity similar to non-health care workers^8^. A study of health behaviors and weight among hospital-based nurses found that the majority of nurses were overweight and obese, and some were not actively involved in weight management behaviors^9^. Physicians may be an exception as they tend to have healthier habits than other health care workers and the general population, except in alcohol abuse^10^.

We conducted this study to evaluate health habits of employees of a large medical center, evaluate time trends of such habits, and compare them to national population data where possible. We also looked at the effect of an onsite, subsidized, wellness facility offering wellness programs and access to exercise equipment.

## Methods

This study was conducted at Mayo Clinic. Mayo Clinic is the largest private employer in the state of MN with 32,347 employees of whom 85.3% are allied health staff, 9.2% are physicians, and 5.5% non-health services staff. In 1995, Mayo Clinic opened a wellness facility, The Dan Abraham Healthy Living Center (DAHLC) in which the aim was to provide members with a comprehensive wellness experience through an extensive range of programs and services. Employees and their adult dependents were eligible for membership and a modest monthly fee was charged ($7/month at the time of opening, currently $31/month). In 2007, DAHLC opened a new state-of-the-art wellness facility in a new building as part of a continuous effort to offer healthy living and wellness programs to employees and their dependents. Programs in the new facility included cooking demonstrations, group fitness classes, individual wellness evaluations and coaching, massage therapy, stress management, and weight loss programs as well as the availability of exercise equipment for individual use. Some of programs required an extra fee.

### Survey description

The 36-item DAHLC Wellness Survey was created in 1996 in collaboration with the Mayo Clinic Survey Research Center. It contained questions on age, height, weight, gender, smoking status, exercise quantity and quality, nutritional habits, and overall quality of life. In 1996, a random sample of Mayo Clinic employees in Rochester, MN, stratified equally by DAHLC membership status, was obtained from the Human Resources Department. In survey years 2007, 2009, and 2010, the random samples were selected from all employees and not stratified by DAHLC membership status. The random samples did not include supplemental employees, temporary employees, or dependents. The method of survey in 1996 was a mail-delivery process, with a subsequent mail-in reminder. Surveys in 2007–10 were sent electronically to be completed online. A subsequent email reminder was sent. No further attempts to include non-respondent employees were undertaken after the second attempt.

**Figure 1:**
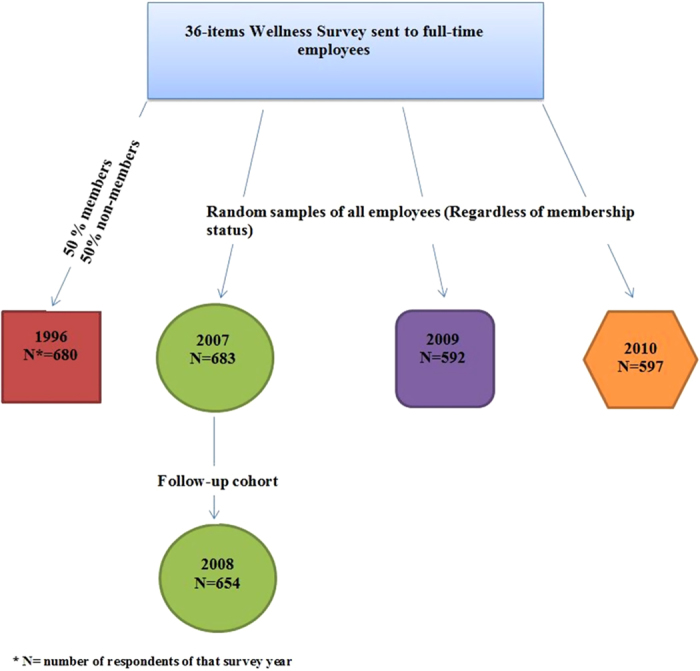
Flow diagram illustrates 5 surveys per year and number of respondents.

The survey in 1996 was conducted one year after the wellness facility opened in 1995. The survey in 2007 was conducted before the new wellness facility opened later that year. The survey in 2008 was conducted after the new facility opened, but before the new incentive program was implemented. The 2008 survey had a slightly different design from other years. The 2007 randomized sample individuals were again sent the survey in 2008 to examine changes in the survey responses in this same cohort. Figure 1 illustrates this process.

For comparisons to the United States general population, data were obtained from the National Health Interview Survey (NHIS)^11^ for smoking and the National Health and Nutrition Examination Survey (NHANES)^12^ and the Behavioral Risk Factor Surveillance System (BRFSS)^13^ for obesity. Definitions from these national surveys were generally comparable to the definition of our survey outcomes except for NHANES, in which obesity was measured. The questions on exercise were slightly different than questions used in national surveys and were not directly comparable. The study was approved by the Institutional Review Board at Mayo Clinic in accordance with relevant guidelines and regulations and surveys were designated exempt from requiring patients consent.

### Incentive program

In 2003, the medical center began encouraging employees to take an online health risk assessment to improve their knowledge of their personal risk profile. In 2008, a new program was implemented that offered monetary incentives to employees for completing the health risk assessment and for participating in programs on physical activity, smoking cessation, nutrition, or weight management through the DAHLC or remotely through online programs or telephonic coaching. The incentive involved accumulating points that could be converted to a reduction ($60 every 6 months) on health insurance premiums.

### Outcomes definition

Subjects were classified as current smokers if they had at least one cigarette per day. BMI was calculated as kg/m^2^ using self-reported height and weight. Regular exercise was defined as engaging in any form of formal exercise at least once/week during their leisure time in the past 4 weeks. The recommended amount of exercise vigorous enough to support cardiovascular fitness was defined as performing exercise at least 3 days per week, at least 15–30 minutes per session, with at least half of each session resulting in shortness of breath.

### Statistical analysis

Continuous data (BMI) were summarized using means and standard deviations, and all remaining data was summarized with frequencies and percentages. The outcomes (obesity, self-reported exercise, achievement of recommended cardiovascular activity, obtaining significant physical activity, and smoking status) were compared between members and non-members within each year using logistic regression models which included predictors for DAHLC membership (yes/no), year (nominally: 1996, 2007, 2008, 2009, 2010), and the interaction between the two in order to estimate the effects separately within year by DAHLC membership status. Each of these regression models utilized generalized estimating equations to account for repeated data between 2007 and 2008 (same cohort). For each model, we reported the odds ratio and 95% confidence interval (CI) comparing the presence vs absence of each outcome for members vs non-members (reference) within year, as well as overall (adjusted for year). To examine the presence of a trend in each of the outcomes over the years (1996–2010), we used Cochran-Mantel-Haenszel (CMH) tests for trend, adjusting for membership status. Further, the obesity rates were compared between employees (stratified by membership) and national estimates with chi-square goodness-of-fit tests.

In order to get an overall estimate of smoking status in the employee population for comparison to national estimates, we also examined the data combining members with nonmembers. To do this, we first adjusted the 1996 estimates for the unequal probability of sampling via weighted analyses. The weights were computed as the inverse of the probability of sampling, rescaled to the total in 1996 (n = 662). Data from 2007–2010 were all given equal weight of 1. Comparisons to national data were performed with a Rao-Scott chi-square goodness-of-fit test for 1996 and with traditional chi-square goodness of fit tests within the remaining years. All analyses were performed SAS, version 9 (Cary, NC). For all analyses, we considered p-values ≤ 0.01 to be statistically significant.

## Results

Overall, response rates ranged from approximately 59% to 68%. Individual year response rates were 68% in 1996 (n = 680, of which 662 were included in the analysis, see below), 68% in 2007 (n = 683), 65% in 2008 (n = 654), 59% in 2009 (n = 592), and 60% in 2010 (n = 597). In 1996, 18 respondents did not list a work location and were not included in the analysis.

### Participants’ characteristics

Between 30–40% of respondents in years 2007–2010 were DAHLC members (50% in 1996 by design). Respondents’ age ranged from <29 years up to >70 years (ages were requested by decades with these age group distributions at either end of the age spectrum). Using the midpoint of each age decade, the average age ranged from 38.9 to 44 among members, and from 40.8 to 44.7 among nonmembers. The majority (71–77%, depending on year and membership status) of respondents were women, which corresponds to the demographic distribution of Mayo Clinic employees. Table 1 details demographics and characteristics of respondents for each survey year.

### Outcomes of interest

#### Obesity

Table 2 outlines BMI classification by survey years stratified by facility membership. The BMI calculated from self-reported weight and height was slightly greater than national data from the BRFSS (self-report), but less than BMI from NHANES (measured). For 2007 (data available from all three sources), the prevalence of obesity was 27.0% among survey respondents (members and non-members combined) compared to 26.3% from the BRFSS and 33.8% from NHANES. Obesity was more prevalent among members of the wellness center (vs. nonmembers) in the initial survey in 1996 (18.8% vs 14.7%) and 2008 (32.4% vs 26.2%), but this was not statistically significant. In other survey years, obesity was more prevalent among nonmembers (1 to 2 percentage points higher) but these differences were not statistically significant. In general, the obesity rate was lowest in 1996 (CMH test for trend, adjusting for membership status: p < 0.0001) as compared to 2007–2010, but there was no significant trend noted from 2007 to 2010, consistent with national trends.

#### Regular exercise and activity at work

Members of the wellness facility had a greater prevalence of regular exercise than nonmembers, with over 2/3 of members and over 1/2 of nonmembers participating in each year (Table 3). In general, the prevalence of regular exercise was significantly higher in 1996 as compared to 2007–2010 (CMH test for trend, adjusting for membership status: p < 0.0001) dropping by 14 percentage points from 1996 to 2007, but there was no significant difference by year from 2007–2010. Within each year, nonmembers were more likely to obtain significant physical activity on the job as compared to members (p = 0.002).

#### Cardiovascular activity

In all years, at least 48% of members and 30% of nonmembers participated in exercise vigorous enough to support cardiovascular fitness (Table 3). Similar to regular exercise, the prevalence of cardiovascular exercise was greater in members than nonmembers at all points in time (p < 0.001), with no significant changes over time in either group.

#### Smoking status

Compared to nonmembers of the wellness facility, members had a lower prevalence of cigarette smoking (year-adjusted OR 0.66, P < 0.001). Employees overall (members and nonmembers combined) had a lower prevalence of cigarette smoking compared to national data in all survey years (P < 0.001) (Table 4).

## Discussion

We conducted a survey of randomly selected employees of a large medical facility to determine the status of health habits, trends over time, and comparisons with national data. We also studied the impact of a worksite wellness facility on employees’ health habits. Employees had a prevalence of obesity that was similar to national data and increased over time, which was also consistent with national data. Employees, however, had a lower prevalence of cigarette smoking than the general population. Membership in the wellness facility was associated with increased levels of exercise and a lower prevalence of cigarette smoking. An incentive program appeared to be associated with trends in short term improvement in health habits among members of the wellness facility.

The relatively low cigarette smoking prevalence of 9.7% in 2010 among healthcare workers at this medical center is a continuation of previous trends. Mayo Clinic implemented a smoke-free policy in 1987. The prevalence of cigarette smoking at Mayo Clinic in 1986, prior to the smoke-free policy was 16.7% and in 1989, 2 years after the smoke-free policy was implemented was 13.8%. By contrast, the prevalence of cigarette smoking in the general population of the US was 28.8% in 1987(34). A Nicotine Dependence Center and other resources have been readily available to medical center employees, which have helped achieve this low prevalence over the years. The lower prevalence of cigarette smoking among members of the wellness facility compared to nonmembers may indicate better overall attention to this particular health habit by members rather than the impact of the wellness facility, as there was no smoking cessation programs located within the facility.

While not statistically significant, there was a trend toward improvement in most health habits among members of the wellness facility in 2009 relative to 2008. This may be a result of the new incentive program implemented in 2008, particularly in view of the consistency of improvement in most health habits. While all employees were eligible for this incentive program, the improvement in health habits was primarily seen in members of the wellness facility. Consistent with the data on smoking, this may indicate greater overall attention to health habits among members of the wellness facility. However, the effect of the incentive program cannot be determined with certainty from these data. This improvement in most health habits in 2009 is unlikely to be the result of the new wellness center in 2007 as there was no improvement seen in 2008, which was after the new center opened. In 2010, all of the health habits regressed back to near 2008 levels, although the incentive program did not change from 2008 to 2009. However, the incentive program was not publicized as much around the medical center in its’ second year in 2009, which may have contributed to a diminished effect. Although the evidence supporting incentive programs is increasing and has shown some success, employers have found implementing such programs can be difficult as reported by a not-for-profit health group coalition^14^.

The interpretation of the changes in BMI and the prevalence of obesity are somewhat more complex. In general, overweight and obesity prevalence estimates were similar to national estimates and increased over time among members and nonmembers of the wellness facility, except for members in 2010, which was consistent with national trends. The prevalence of obesity, however, increased among members of the wellness facility in 2008 and decreased among both members and nonmembers in 2010. Also, in 1996 and 2008, members of the wellness facility had a higher prevalence of obesity compared to nonmembers, which was reversed in other years. Possible explanations for these finding are that first, many employees who were slightly heavier sought to lose weight when the wellness facility first opened in 1995 and when the new wellness facility opened in 2007, whereas in other years the effect of attending the wellness center on the members’ weight (ie, decreasing) may have been more apparent. In addition, the decreasing prevalence of obesity among both members and nonmembers in 2010 and the decreased mean BMI among members in 2010 may be due to the combined effects of the new wellness facility and the incentive program in 2008. However, all of these changes may be mere trends and causation cannot be determined from the data. Additionally, these outcomes were self-reported and this may present a limitation to better interpret these results.

The questions on exercise and cardiovascular exercise were first used in the 1996 survey. To maintain consistency and internal comparability, these questions were maintained through the subsequent surveys. However, this limited comparability with national estimates as the questions in these surveys was slightly different than those used in national surveys.

Workers in healthcare settings may have unique stressors such as unusual work hours, issues with understaffing, or working in a litigious environment that limits their pursuit of exercise or healthy lifestyle habits^15^. On the other hand, workers in this setting may have more access to educational health information, medical recommendations, and treatment programs, which may explain the lower prevalence of cigarette smoking compared to national data.

Physicians are a group within the health care sector that generally have healthier habits than other health care workers and the general public including a lower prevalence of smoking, obesity, diabetes, hypertension, and hyperlipidemia; and higher exercise prevalence^16^,^17^,^18^,^19^,^20^,^21^. However, they may have increased rate of alcohol abuse and other areas of substance abuse^10^,^22^. Occupation was not requested in the survey so secondary analysis of results based on specific occupation was not possible.

The USDHHS Healthy People 2020 objectives suggest establishing comprehensive wellness programs that incorporate five attributes: health education, links to related employee services, supportive physical and social environments for health improvement, integration of health promotion into the organization’s culture, and employee screenings with adequate treatment and follow-up^3^. According to the Society for Human Resources Management (SHRM), approximately 60% of all United States companies had implemented some type of a wellness program in 2011^23^. However, only 6.9% of corporations surveyed in the 2004 National Worksite Health Promotion Survey possessed all five of these attributes^24^. SHRM also reported there was only a 2% increase in wellness programs offered by companies from 2008–2011^23^.

The health care industry is currently the largest industry in the United States, employing approximately 14 million in 2006 and projected to add an additional 3.5 million positions between the years 2010 and 2020^25^. Therefore, improving the health of this segment of the population is important and should lead to cost savings^26^. Furthermore, workers in healthcare may serve as role models, potentially propagating healthy lifestyle practices to other segments of the population and throughout their community.

## Conclusions

Healthcare workers in this large medical center reported less cigarette smoking compared to the general population, while the prevalence of obesity was similar. Membership in a worksite wellness facility was associated with increased levels of exercise and a lower prevalence of cigarette smoking.

## Additional Information

**How to cite this article**: Dabrh, A. M. A. *et al.* Health Habits of Employees in a Large Medical Center: Time Trends and Impact of a Worksite Wellness Facility. *Sci. Rep.* **6**, 20804; doi: 10.1038/srep20804 (2016).

## Figures and Tables

**Table 1:** Respondent characteristics by year, stratified by Wellness facility membership.

	1996	2007	2008	2009	2010
		(Single cohort)*			
Overall, N	662	683	654	592	597
Members					
N (% of Overall)†	332 (50.1)	210 (30.7)	254 (38.8)	226 (38.2)	207 (34.7)
Gender, n (%)					
Male	78 (23.6)	56 (27.1)	68 (27)	60 (26.8)	53 (25.9)
Female	253 (76.4)	151 (72.9)	184 (73)	164 (73.2)	152 (74.1)
Age^‡^					
Average, SD	38.9 (9.2)	40.5 (11.0)	42.8 (11.0)	42.5 (12.1)	44.5 (12.3)
Non-Members					
N (% of Overall)†	330 (49.8)	473 (69.3)	400 (61.2)	366 (61.8)	390 (65.3)
Gender, n (%)					
Male	95 (28.8)	117 (24.8)	104 (26.4)	84 (23.2)	95 (24.7)
Female	235 (71.2)	355 (75.2)	290 (73.6)	278 (76.8)	290 (75.3)
Age‡					
Average, SD	40.8 (9.7)	44.0 (10.8)	46.0 (10.8)	46.0 (10.7)	44.7 (11.6)

^*^Survey sent to the same 1000 individuals in 2007 and 2008, though, not everyone responded to each of the two surveys.

^†^Numbers not totaling to the overall N indicate missing data; 1996 had 50% members and non-members by design.

^‡^Age assessed categorically by decade. Average calculated based on the midpoint of each decade.

**Table 2:** Comparison of BMI by Wellness facility membership and national estimates.

Outcome	Group	1996	2007	2008	2009	2010	Overall OR
			(Single cohort)†				
BMI							
<25, %	Members	51.2%	41%	37.6%	35.6%	41.5%	
25 to 30, %		29.9%	32.2%	30%	36.5%	32%	
> = 30, %		18.8%	26.8%	32.4%	27.9%	26.5%	
<25, %	Nonmembers	50.9%	39.5%	38.9%	39.6%	36.6%	
25 to 30, %		34.4%	33.5%	34.9%	30.2%	35.8%	
> = 30, %		14.7%	27.1%	26.2%	30.2%	27.5%	
	OR (95% CI)^‡^	1.28 (0.91, 1.81)	1.07 (0.89, 1.27)	1.17 (0.99, 1.38)	0.92 (0.71, 1.20)	0.98 (0.76, 1.27)	1.08 (0.97, 1.20)
	*P-value*‡	0.27	0.93	0.20	0.29	0.49	0.17
<25, %	National§	47.8%	37%	36.6%	36%	35.5%	
25 to 30, %		35.4%	36.7%	36.5%	36.2%	36.2%	
> = 30, %		16.8%	26.3%	26.6%	26.9%	27.5%	
P-values comparing to National estimates	*Member vs. National*	0.12	0.36	0.05	0.95	0.21	
	*Non-member vs. National*	0.45	0.35	0.66	0.05	0.93	

^†^Survey sent to the same 1000 individuals in 2007 and 2008, though, not everyone responded to each of the two surveys.

^‡^Odds ratios (and p-value below each OR) compare for members versus non-members (reference). Odds ratios are comparing odds of obesity between groups. The odds ratio in the last column is comparing the odds of obesity overall between members and non-members (reference), adjusted for year.

^§^Data from the Behavioral Risk Factor Surveillance Survey^12^.

**Table 3:** Comparison of selected respondent characteristics between Wellness facility members versus non-members.

Outcome	Group	1996	2007	2008	2009	2010	Overall OR
			(Single cohort)*				
Engaged in regular formal exercise during last 4 weeks, %	Member	80.9%	66.8%	66.8%	72.3%	67.8%	
	Non-Member	69.1%	54.8%	54.2%	51.6%	50.4%	
	OR (95% CI)†	1.17 (1.07, 1.28)	1.21 (1.07, 1.37)	1.21 (1.07, 1.37)	1.40 (1.23, 1.59)	1.34 (1.17, 1.54)	1.25 (1.18, 1.32)
	*P-value*†	<0.001	0.003	0.001	<0.001	<0.001	<0.001
Obtains significant physical activity at job, %	Member	40.9%	32.7%	31.5%	36.3%	26.2%	
	Non-Member	49.4%	34.5%	38.3%	40.4%	36.8%	
	OR (95% CI)^†^	0.83 (0.70, 0.98)	0.99 (0.83, 1.19)	0.90 (0.75, 1.07)	0.90 (0.73, 1.11)	0.72 (0.56, 0.92)	0.86 (0.79, 0.95)
	*P-value*†	0.03	0.65	0.08	0.31	0.009	0.002
Recommended cardiovascular activity, %	Member	51.2%	48.6%	47.6%	56.2%	52.7%	
	Non-Member	34%	30.7%	33.3%	30.6%	33.1%	
	OR (95% CI)†	1.50 (1.25, 1.81)	1.58 (1.31, 1.90)	1.35 (1.13, 1.62)	1.84 (1.51, 2.23)	1.60 (1.33, 1.94)	1.57 (1.44, 1.71)
	*P-value*†	<0.001	<0.001	<0.001	<0.001	<0.001	<0.001
Current smoker, %	Member	7.8%	7.1%	7.1%	4.4%	7.2%	
	Non-Member	12.1%	10.1%	8.5%	10.4%	11%	
	OR (95% CI)†	0.65 (0.40, 1.03)	0.82 (0.53, 1.26)	0.71 (0.45, 1.13)	0.43 (0.22, 0.84)	0.67 (0.39, 1.15)	0.66 (0.51, 0.83)
	*P-value*†	0.07	0.21	0.51	0.01	0.14	<0.001

^*^Survey sent to the same 1000 individuals in 2007 and 2008, though, not everyone responded to each of the two surveys.

^†^Odds ratios (and p-value below each OR) compare for members versus non-members (reference). The odds ratio in the last column is comparing the odds of each outcome overall between members versus non-members (reference), adjusted for year.

**Table 4:** Comparison of cigarette smoking prevalence between employees (members and non-members combined) and national data.

Outcome	Group	1996†	2007	2008	2009	2010
			(Single cohort)*			
Current smoker, %	Employees	11.3%	9.2%	8%	8.1%	9.7%
	National§	23.5%	19.7%	18.4%	17.9%	17.3%
	*P-value*	<0.001	<0.001	<0.001	<0.001	<0.001

NA = Not available.

^*^Survey sent to the same 1000 individuals in 2007 and 2008, though, not everyone responded to each of the two surveys.

^†^All estimates and analyses for 1996 are weighted to account for unequal probability of sampling (members were oversampled). See methods section for details.

^§^The National Health Interview Survey (NHIS)^11^.
